# T-Cell Subsets Predict Mortality in Malnourished Zambian Adults Initiating Antiretroviral Therapy

**DOI:** 10.1371/journal.pone.0129928

**Published:** 2015-06-17

**Authors:** Caroline C. Chisenga, Suzanne Filteau, Joshua Siame, Molly Chisenga, Andrew J. Prendergast, Paul Kelly

**Affiliations:** 1 Tropical Gastroenterology and Nutrition group, University of Zambia School of Medicine, Lusaka, Zambia; 2 NUSTART project, University Teaching Hospital, Lusaka, Zambia; 3 London School of Hygiene & Tropical Medicine, London, United Kingdom; 4 Barts & The London School of Medicine, Queen Mary University of London, London, United Kingdom; The University of Melbourne, AUSTRALIA

## Abstract

**Objective:**

To estimate the prognostic value of T-cell subsets in Zambian patients initiating antiretroviral therapy (ART), and to assess the impact of a nutritional intervention on T-cell subsets.

**Methods:**

This was a sub-study of a randomised clinical trial of a nutritional intervention for malnourished adults initiating ART. Participants in a randomised controlled trial (NUSTART trial) were enrolled between April and December 2012. Participants received lipid-based nutritional supplement either with or without additional vitamins and minerals. Immunophenotyping was undertaken at baseline and, in survivors, after 12 weeks of ART to characterize T-cell subsets using the markers CD3, CD4, CD8, CD45RA, CCR7, CD28, CD57, CD31, α4β7, Ki67, CD25 and HLA-DR. Univariate and multivariate survival analysis was performed, and responses to treatment were analysed using the Wicoxon rank-sum test.

**Results:**

Among 181 adults, 36 (20%) died by 12 weeks after starting ART. In univariate analysis, patients who died had fewer proliferating, more naïve and fewer gut homing CD4^+^ T-cells compared to survivors; and more senescent and fewer proliferating CD8^+^ T-cells. In a multivariate Cox regression model high naïve CD4^+^, low proliferating CD4^+^, high senescent CD8^+^ and low proliferating CD8^+^ subsets were independently associated with increased risk of death. Recent CD4^+^ thymic emigrants increased less between recruitment and 12 weeks of ART in the intervention group compared to the control group.

**Conclusions:**

Specific CD4^+^ T-cell subsets are of considerable prognostic significance for patients initiating ART in Zambia, but only thymic output responded to this nutritional intervention.

## Introduction

Although the annual number of AIDS-related deaths has declined from 2.3 (2.1–2.6) million in 2005, to 1.6 (1.4–1.9) million in 2012 [1] with the expansion of antiretroviral therapy (ART) provision in sub-Saharan Africa, studies consistently report high mortality during the first 6 months following ART initiation [2,3]. The total CD4^+^ T-cell count at ART initiation has important prognostic implications [4,5]. However, there is very little evidence regarding the prognostic value of specific T-cell subsets on early mortality [6,7].

Studies that have evaluated baseline T-cell subsets have reported that, in HIV-infected untreated adults, more activated CD8^+^ T-cells [7], and fewer CD28^+^ CD4^+^ T-cells [8] or naïve CD4^+^ T-cells independently predicted mortality [9]. In recently HIV-infected and untreated adults, low proportions of senescent (CD57^+^) CD4^+^ T-cells were associated with 5-fold higher odds of mortality [8]. In Zambian children, more CD8^+^ effector T-cells with fewer CD4^+^ central memory T-cells was associated with increased risk of mortality [6].

Several studies in Europe and Africa have shown associations between early mortality and low body mass index and deficiencies in micronutrients at ART initiation [10,11,12,13,14]. However, randomised controlled trials of nutritional supplements, conducted in both children and adults, have not shown a clear benefit on mortality [15,16,17]. Researchers have also reported an insignificant increase in CD4^+^ cell count following macronutrient supplementation [15]. However, there is emerging evidence that micronutrient supplementation increases absolute CD4^+^ cell counts [16,17].

This study was designed to investigate, first, whether specific T-cell subsets predict mortality in a cohort of HIV-infected Zambian adults initiating ART and, second, whether a nutritional intervention impacts T-cell subsets in survivors.

## Methods

### NUSTART Trial Design and Population

We report a sub-study of a randomized controlled trial (Nutritional Support for Africans starting ART, NUSTART) of a vitamin and mineral intervention for HIV-infected adults initiating ART, full details of which are reported elsewhere [17]. Participants were recruited in Lusaka, Zambia and Mwanza, Tanzania from August 2011 to December 2013. NUSTART was a phase III individually randomised controlled trial which compared, in a two-stage protocol, vitamins and minerals in a lipid-based nutritional supplement (LNS-VM) with unfortified LNS (LNS) given from recruitment (referral for ART) until 6 weeks after starting ART (S1 Table). In the first stage, from referral until 2 weeks after ART initiation, the active and control supplements were given with minimal calories i.e. 30 g/day, 100 kcal/day, only as the lipid-based carrier, then from 2–6 weeks after ART initiation, patients were given 250 g/day, in two 125 g sachets, comprising 1360 kcal/day in a calorie-rich supplement. Compliance to ART was high: 95% of patients took at least 95% of medication [17]. The NUSTART trial is registered as PACTR201106000300631.

### Sub-study Design and Patient Profile

All adults recruited into the NUSTART trial in Lusaka between April and December 2012 were eligible for this sub-study. Enrolment occurred at 6 different public clinics during routine assessment for ART eligibility. Following enrolment, a baseline questionnaire was administered and anthropometric and clinical data collected. All adults eligible for ART (CD4 count <350 cells/μL and/or WHO stage III or stage IV disease) with body mass index (BMI) of <18.5 kg/m^2^ were eligible for the NUSTART trial. Over 90% of NUSTART trial patients in Lusaka started a first-line regimen of tenofovir, emtricitabine and efavirenz, with cotrimoxazole.

### Ascertainment of outcome and follow-up

The primary endpoint for the main NUSTART trial was mortality between the time of recruitment (referral for ART) and 12 weeks after starting ART. NUSTART study participants were followed up weekly before starting ART and at weeks 2, 4, 6, 8 and 12 after ART initiation. Whether the patient was still alive, and the date of death if not, were ascertained by phone contact or home visit if the patient failed to return to the next scheduled visit. Patients were evaluated at 12 weeks following ART initiation and immunophenotyping was carried out on baseline and 12 week blood samples.

### Grip Strength

The maximum isometric strength of the hand and forearm muscles was measured using a grip strength dynamometer (Takei, Japan). The handle for the machine was positioned so there was a 90-degree angle in the second joint of the index finger. The participant then squeezed the handle as hard as possible once for four times in the following order: right-left-right-left. The instrument calculated the average of these measurements.

### Samples

At recruitment and at week 12 following initiation of ART, 3–5 ml of peripheral blood was collected into an EDTA tube for lymphocyte analysis. Blood samples reached the laboratory within 1h of collection and were subdivided for haemoglobin and immunophenotyping. Haemoglobin was measured on a Sysmex xt 4000i automated haematology analyser (Sysmex Corporation, Kobe, Japan). For immunophenotyping, samples were stained and analysed where possible within 2 hours by flow cytometry, after being suspended in paraformaldehyde; where this was not possible, stained samples were suspended in BD CellFix and flow cytometry undertaken the following day.

### Laboratory assays

Immunophenotyping was done using fluorescent-labelled monoclonal antibodies to differentiate T-cell subsets, using CD3 APC-H7 (clone SK7), CD4 FITC (clone RPA-T4) or CD4 PerCP (clone L200), CD8 FITC (clone 3B5), HLA-DR APC (clone TU36), Ki67 FITC (clone B56), CCR7 PE (clone 150503), CD25 PE-Cy7 (clone M-A251), Alpha-4 FITC (clone 9F10), Beta-7 APC (clone FIB504), CD57 APC (clone NK-1), CD27 APC or PE-Cy7 (clone M-T271), CD28 FITC or PE (clone CD28.2), CD31 PE (clone WM59), CD45RA PE-Cy7 (clone HI100) and 7-AAD PerCP (all from BD Biosciences, Gauteng, South Africa) in panels, as outlined below. Data were acquired on a six-colour FACSverse flow cytometer (Becton Dickinson, San Jose, U.S.A.). 100 μL of whole blood was stained, red cells were lysed, then cells were washed and resuspended in 250 μL paraformaldehyde or 10% cell fix before flow cytometric analysis. Intracellular Ki-67 staining was carried out after resuspension in 1X Perm solution (BD) for nuclear membrane permeabilisation. Between 50,000–100,000 lymphocyte events were acquired per tube and fluorescence-minus-one (FMO) controls were used to set all gates. Analysis of markers was carried out using BD FACSuite^TM^ software v1.0.2.

### CD4^+^ and CD8^+^ T-cell Subset Definition

Lymphocytes were gated on CD3^+^ and CD4^+^ or CD8^+^ to identify T-cell populations, and subsets distinguished as follows: naïve (CCR7^+^CD45RA^+^), central memory (CCR7^+^CD45RA^-^), effector memory (CCR7^-^CD45RA^-^), effector (CD45RA^+^CCR7^-^), gut homing (α4^+^β7^+^), activated gut homing (α4^+^β7^+^CD25^+^), recent thymic emigrants (CD45RA^+^CD31^+^), activated (HLA-DR^+^), proliferating (Ki67^+^), activated and proliferating (Ki67^+^ HLA-DR^+^), or senescent (CD57^+^).

### Data Analysis

T-cell markers were found to be non-parametrically distributed using the Shapiro-Wilk W’ test for normality, so results were expressed as medians and interquartile ranges, and variables compared using the Mann-Whitney U test, or the Wilcoxon rank sum test for paired variables. *P* values less than 0.05 were considered statistically significant. For analysis of predictors of mortality, all cellular subsets plus total CD4^+^ and CD8^+^ count, BMI, haemoglobin, sex and grip strength were included in univariate analysis and *p* values calculated using Fisher’s exact test for unpaired variables. In multivariate analysis, all cellular subsets including total CD4^+^ and CD8^+^ count, BMI, haemoglobin, sex and grip strength were included and hazard ratios and *p* values determined using the Cox regression model. For both univariate and multivariate analysis, T-cell markers were dichotomised around the median (except for total CD4^+^ count which was dichotomised around 100 cells/μL, rather than the median since it is a standard cut-off, also used in another study [18], and which resulted in close to equal numbers in both groups), and the risk and hazard ratios for mortality show the effect of the upper half compared to the lower half. To generate Kaplan-Meier curves, low numbers of proliferating CD4^+^ cells and high numbers of naive CD4^+^ cells were used as independent variables and their effect assessed using the log-rank test. All data were analysed using STATA version 13 (StataCorp LP, College Station, Texas, USA).

### Study Approval

Ethical approval for the NUSTART trial was granted by the London School of Hygiene and Tropical Medicine, the University of Zambia Biomedical Research Ethics Committee, and the Medical Research Coordinating Committee of National Institute for Medical Research, Tanzania. The University of Zambia Biomedical Research Ethics Committee approved the current sub-study (Ref. No. 009-01-11). Each participant provided written or thumbprint informed consent for participation in the trial.

## Results

### Characteristics of study participants

**Fig 1:**
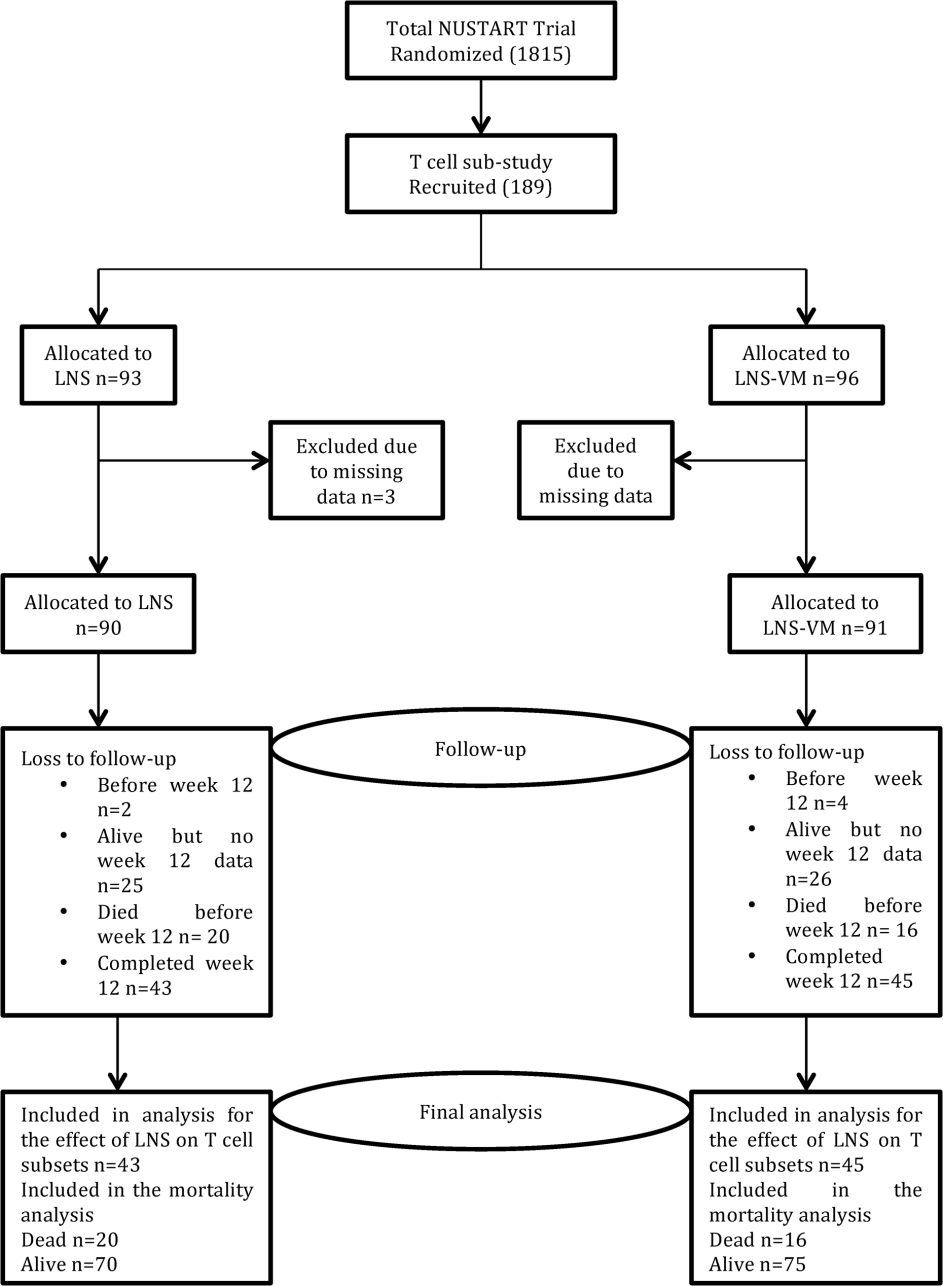
Flow of participants through the study.

A total of 189 HIV-infected adult men and women were enrolled in this sub-study and randomised to either LNS or LNS-VM. Participants were excluded from analysis if some baseline CD4^+^ and CD8^+^ T-cell panel data were missing (n = 3 LNS and n = 5 LNS-VM). Of the remaining 181 participants, 90 were in the LNS and 91 in the LNS-VM group (Fig 1). Groups randomised to LNS and LNS-VM were very similar (Table 1). Those who died had lower total CD4^+^ cell count, lower grip strength and a small difference in BMI than survivors (Table 1). Among all participants in this sub study, the median CD4 count at week 0 (enrolment) was 98 (IQR 64, 211) and at week 12 (primary endpoint) was 259 (IQR 166, 389) (*p*<0.0001).

**Table 1:** Baseline clinical and nutritional characteristics of HIV-infected adult Zambian men and women^1^.

Survivor status				Clinical trial	
Variable	Died (n = 36)	Survived (n = 145)	*P*	LNS (n = 90)	LNS-VM (n = 91)
Age (years)	36 (34, 42)	35 (30, 41)	0.10	36 (33, 42)	36 (31, 42)
Male	20 (56%)	82 (57%)	0.53	47 (51%)	60 (63%)
Haemoglobin (g/dl)	10.6 (9.2, 12.3)	10.3 (8.7, 11.6)	0.45	10.0 (8.5, 12.0)	10.7 (9.5, 11.4)
Total CD4 (cells/μl)	73 (39, 145)	125 (58, 234)	0.01	119 (66, 264)	87 (55, 205)
Total CD8 (cells/μl)	893 (516, 1270)	856 (583, 1370)	0.51	962 (596, 1304)	974 (674, 1542)
BMI (kg/m^2^)	16.4 (15.6, 17.2)	16.7 (15.8, 17.8)	0.07	17.0 (16.0, 18.1)	16.6 (15.8, 17.6)
Grip strength (kg)	14.5 (8.7, 16.8)	20.2 (15.4, 24.9)	<0.0001	20.2 (14.9, 26.6)	20.0 (15.5, 24.1)
On TB treatment	6 (17%)	49 (34%)	0.045	19 (21%)	36 (39%)
Education					
- No education	5 (14%)	19 (13%)	0.85	14 (15%)	10 (13%)
- Primary	18 (50%)	62 (43%)		37 (42%)	42 (44%)
- Secondary	12 (33%)	59 (41%)		36 (40%)	37 (38%)
- Tertiary	1 (3%)	4 (3%)		3 (3%)	2 (5%)
Occupation					
- Employed	14 (39%)	82 (57%)	0.17	47 (51%)	49 (52%)
Marital status					
- Single	18 (50%)	57 (39%)	0.45	32 (36%)	35 (38%)
- Married	16 (44%)	74 (51%)		52 (55%)	47 (50%)
- Widowed	2 (6%)	14 (10%)		6 (9%)	9 (12%)

^**1**^ Mann–Whitney U-tests were used for non-parametric variables and t tests for parametric variables. Continuous characteristics are expressed as median (interquartile range) and dichotomous characteristics are expressed as n (%). g/dl = grams per decilitre; BMI = body mass index; LNS = lipid-based nutritional supplement; LNS-VM = LNS with added vitamins and minerals.

### Mortality between referral for ART and 12 weeks after starting ART

The first aim of this study was to understand whether specific T-cell subsets predict mortality in a cohort of HIV-infected Zambian adults initiating ART. Of 181 participants, 36 (20%) died at median (IQR) 5.7 (3.5, 10.1) weeks after ART initiation; 145 (80%) survived through 12 weeks after initiating ART. Patients who died, compared to those who survived, had a lower total CD4^+^ T cell count and had more naïve, more central memory, fewer effector memory, fewer proliferating, fewer gut-homing and fewer gut-homing activated CD4^+^ T-cells (Table 2). Patients who died, compared to those who survived, had more senescent, fewer effector or effector memory, fewer proliferating, more activated proliferating and more activated gut-homing CD8^+^ T-cells (Table 2).

**Table 2:** Baseline CD4 and CD8 T cell subsets among those who survived or died and those in LNS or LNS-VM between referral for ART and 12 weeks after starting Antiretroviral Therapy.

Survivor status (n = 181)				Clinical trial (n = 88)	
	Died (n = 36)	Survived (n = 145)		LNS (n = 43)	LNS-VM (n = 45)
**CD4 subsets**					
**Variable (cells/uL)**	**Median (IQR)**	**Median (IQR)**	***P***	**Median (IQR)**	**Median (IQR)**
Total count	73 (39,145)	125 (58, 234)	0.01	119 (66, 243)	87 (55, 205)
Thymic emigrants	9 (2, 37)	6 (2, 32)	0.52	7 (2, 32)	6 (2.5, 32.5)
Senescent	4 (0, 21)	7 (2, 17)	0.28	0.5 (0, 9)	2 (0, 6)
Naïve	10 (1, 79)	2 (0, 11)	0.01	2 (0, 5)	6 (2.5, 32.5)
Central memory	5 (0, 25)	1 (0, 4)	0.02	1 (0, 6)	1 (0, 4.5)
Effector memory	46 (26, 75)	62 (28, 137)	0.045	60.5 (32, 172)	62 (18, 127)
Effector	12 (4, 27)	15 (3, 43)	0.45	21.5 (6, 43)	9 (3, 32)
Activated	1 (0, 4)	1 (0, 4)	0.74	2 (0, 5)	2 (1, 9)
Proliferating	12 (7, 22)	28 (12, 80)	<0.0001	65 (13, 183)	39 (18, 137)
Proliferating & activated	1 (0, 2)	1 (0, 4)	0.82	0 (0, 3)	1 (0, 8)
Gut homing	23 (6, 45)	42 (19, 95)	0.001	65 (28, 102)	40 (23, 78)
Gut homing & activated	6 (1, 17)	18 (8, 40)	<0.0001	15 (7, 27)	11 (4, 25)
**CD8 subsets**					
Total count	893 (516,1270)	856 (583,1370)	0.51	962 (596, 1304)	974 (674, 1542)
Thymic emigrants	354 (220, 548)	302 (161, 546)	0.34	279 (139, 542)	277 (155, 444)
Senescent	251 (122, 374)	101 (15, 320)	0.01	148 (0, 444)	157 (0, 491)
Naïve	11 (2, 51)	29 (7, 56)	0.10	44 (22, 125)	36 (8, 55)
Central memory	2 (0, 15)	1 (0, 13)	0.67	0 (0, 10)	0 (0, 14)
Effector memory	41 (6, 84)	102 (13, 279)	0.01	187 (51, 381)	231 (141, 411)
Effector	131 (34, 251)	268 (24, 707)	0.01	598 (252, 978)	589 (394, 1060)
Activated	73 (22, 186)	39 (7, 214)	0.33	78 (101, 312)	88 (6, 464)
Proliferating	1 (0, 7)	19 (1, 419)	<0.0001	216 (0, 626)	324 (0, 704)
Proliferating & activated	205 (53, 581)	63 (16, 278)	0.004	24 (4, 128)	63 (13, 249)
Gut homing	378 (211, 607)	450 (281, 786)	0.13	505 (292, 804)	513 (330, 914)
Gut homing & activated	20 (9, 67)	8 (1, 20)	0.001	6 (0, 14)	4 (0, 12)

*p-values determined using Mann–Whitney U-tests. Subsets of CD4^+^ T cells are presented as median (interquartile range) absolute cells/μL and defined as gut homing: α4^+^β7^+^, homing activated: α4^+^7^+^CD25^+^, recent thymic emigrants: CD45RA^+^CD31^+^, senescent: CD57^+^, naive: CCR7^+^CD45RA^+^, central memory: CCR7^+^CD45RA^-^, effector memory: CCR7^-^CD45RA^-^, effector: CCR7^-^CD45RA^+^, activated: HLA-DR^+^, proliferating and activated HLD-DR^+^Ki 67^+^; and proliferating: Ki67^+^.

In univariate analysis of dichotomised variables, fewer total CD4^+^, more senescent, or naïve, fewer proliferating, gut homing or gut homing activated CD4^+^ T-cells were all predictors of death (Table 3). More senescent, activated, activated proliferating, or activated gut homing and fewer effector, effector memory or proliferating, CD8^+^ T-cells also predicted death (Table 3).

**Table 3:** Immunological Risk Factors for Death Within 12 Weeks of Initiating Antiretroviral Therapy.

	Univariate	Multivariate
Variable	Unadjusted HR (95% CI)	Adjusted HR (95% CI)
CD4 Total	0.51 (0.27 – 0.95) *	
CD4 Thymic emigrants	0.89 (0.49–1.59)	
CD4 Senescent	1.97 (1.03–3.76) *	
CD4 Naïve	2.25 (1.78–4.29) *	2.88 (1.39 – 5.97) *
CD4 Central memory	1.46 (0.81–2.63)	
CD4 Effector memory	0.49 (0.27–0.94)	
CD4 Effector	0.70 (0.38–1.27)	
CD4 Activated	0.68 (0.36 – 1.30)	
CD4 proliferation	0.29 (0.14 – 0.60) *	0.43 (0.19 – 0.98) *
CD4 Proliferating & activated	0.68 (0.36 – 1.30)	
CD4 Gut homing	0.39 (0.20–0.76) *	
CD4 Gut homing activated	0.39 (0.20–0.76) *	
CD8 Total	1.13 (0.63 – 2.03)	
CD8 Thymic emigrants	1.42 (0.78–2.57)	
CD8 Senescent	3.03 (1.51–6.08) *	6.62 (2.94 – 14.88) *
CD8 Naïve	0.64 (0.35–1.18)	
CD8 Central memory	1.16 (0.64–2.08)	
CD8 Effector memory	0.51 (0.27–0.95) *	
CD8 Effector	0.52 (0.28–0.98) *	
CD8 Activated	1.83 (0.99–3.38) *	
CD8 Proliferation	0.25 (0.12–0.54) *	0.12 (0.05 – 0.28) *
CD8 Proliferating & activated	1.79 (0.97–3.31) *	
CD8 Gut homing	0.72 (0.40–1.31)	
CD8 Gut homing and activated	2.07 (1.10–3.88) *	
BMI	0.66 (0.37 – 1.19)	
Hb	0.38 (0.14 – 1.07)	
Sex	0.97 (0.54 – 1.74)	
Grip strength	0.23 (0.11 – 0.50) *	0.23 (0.10 – 0.53)*

All variables were calculated using absolute numbers. 36 participants died within 12 weeks of follow up. All variables were dichotomized; univariate logistic regression models were constructed comparing the upper half of the distribution with the lower half. Multivariate analysis was done using a Cox regression model, and was adjusted for, total CD4 and CD8 counts plus BMI, haemoglobin, sex and grip strength. HR = hazard ratio

* = significant CI (confidence interval).

**Fig 2:**
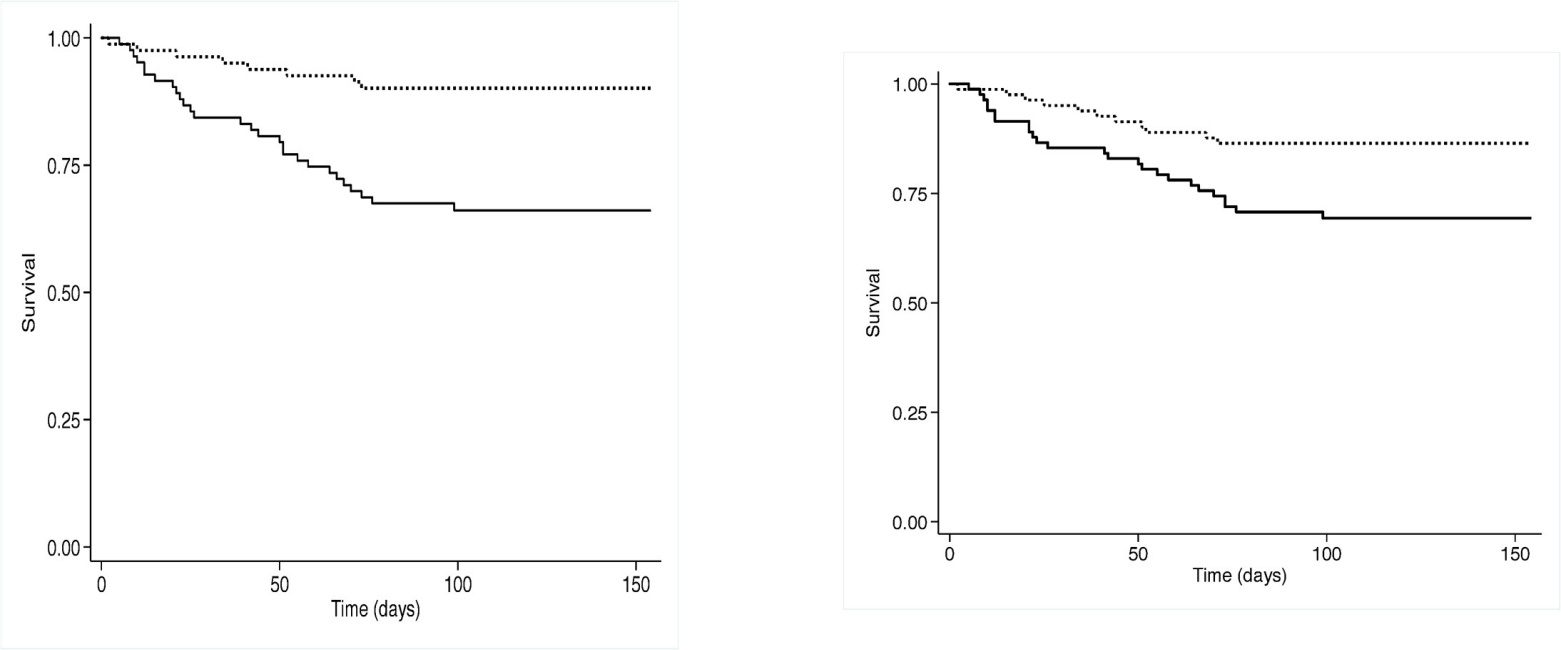
Survival among HIV-infected adults initiating antiretroviral therapy according to proliferating and naïve CD4 cell counts. (Left) Kaplan-Meier plot of survival over time among HIV-infected adults with high (dashed line) or low (solid line) absolute number of CD4^+^ cells expressing the proliferation of marker Ki67 (*p* = 0.0003 using log rank test). (Right) Kaplan-Meier plot of survival over time among HIV-infected adults with low (dashed line) or high (solid line) absolute number of CD4^+^ cells expressing the naïve markers CD45RA and CCR7 (*p*<0.001 using the log rank test).

Using a Cox regression model that included all baseline cellular CD4^+^ and CD8^+^ subsets plus BMI, haemoglobin, grip strength and sex, higher numbers of circulating naïve CD4^+^ and senescent CD8^+^ cells were associated with increased mortality, whilst higher numbers of proliferating CD4^+^ and CD8^+^ cells were associated with reduced risk of mortality by 12 weeks (Table 3). The effect of high versus low CD4^+^ T-cell proliferation at baseline on subsequent survival (*p* = 0.0003 using the log rank test) is illustrated by the Kaplan-Meier curve in Fig 2 (left panel), and the effect of high vs low naïve T-cell numbers (*p*<0.001) is shown in Fig 2 (right panel).

### Survivors randomised to receiving ART with/without vitamin and mineral

The second aim of this study was to investigate the effect of the nutritional intervention on T-cell subsets after 12 weeks. Of the 145 survivors, 57 withdrew and did not have follow-up samples collected, so 88 who completed their 12 week follow-up visit were included in this part of the analysis. Of all the T-cell subsets analysed (Table 4), only the increase in CD4^+^ recent thymic emigrants was significantly different in the participants allocated to receive LNS-VM compared to LNS.

**Table 4:** Changes in T cell subset markers in patients receiving supplements with (LNS-VM) or without (LNS) additional vitamins and minerals.

	CD4			CD8		
	Change in LNS-VM group (N = 45)	Change in LNS group (N = 43)		Change in LNS-VM group (N = 45)	Change in LNS group (N = 43)	
Variable	Median (IQR)	Median (IQR)	*P*	Median (IQR)	Median (IQR)	*P*
Total count	126 (82, 210)	122 (81, 204)	0.98	-27 (-284, 173)	7 (-300, 213)	0.83
Thymic emigrants	8.5 (1.5, 41)	29 (7, 61)	0.01	144 (-27, 306)	87 (20, 221)	0.88
Senescent	-1 (-11, 8)	-0.5 (-10, 11)	0.93	44 (-112, 191)	10 (-90, 194)	0.81
Naïve	2 (-2, 28)	7 (0, 30)	0.27	46 (0, 221)	32 (-19, 380)	0.90
Central memory	5 (-2, 27)	6.5 (1, 25)	0.40	11 (0, 66)	20 (0, 79)	0.34
Effector memory	91 (39, 145)	64 (25, 169)	0.45	-71 (-141, 30)	-65 (228,57)	0.81
Effector	7 (-1, 32)	3 (-10, 30)	0.27	-130 (-299, 72)	-78 (-542, 133)	0.80
Activated	4 (0, 16)	4 (-1, 17)	0.73	194 (-31, 431)	196 (52, 371)	0.94
Proliferation	50 (-30, 126)	62 (-6, 106)	0.69	377 (-98, 877)	350 (25, 782)	0.81
Proliferating & activated	1 (-2, 8)	2 (-1, 10)	0.48	25 (-16, 174)	41 (-16, 123)	0.72
Gut homing	-39 (-78, -22)	-64 (-102, -27)	0.33	-51 (-186, 55)	-65 (-273, 62)	0.93
Gut homing & activated	-8 (-15, -2)	-11 (-20, 0)	0.62	0 (-4, 4)	0 (-6, 3)	0.90

*p-values determined using Wilcoxon rank-sum tests. Changes in subsets of CD4^+^ and CD8^+^ T cells are presented as median (interquartile range absolute cells/μL and defined as gut homing: α4^+^β7^+^, homing activated: α4^+^β7^+^CD25^+^, recent thymic emigrants: CD45RA^+^CD31^+^, senescent: CD57^+^, naive: CCR7^+^CD45RA^+^, central memory: CCR7^+^CD45RA^-^, effector memory: CCR7^-^CD45RA^-^, effector: CCR7^-^CD45RA^+^, activated: HLA-DR^+^ and proliferating and activated: HLD-DR^+^Ki 67^+^; proliferating: Ki67^+^.

## Discussion

Consistent with previous reports from sub-Saharan Africa [4,5], we found high mortality (20%) within 12 weeks of initiating ART among a cohort of malnourished HIV-infected Zambian adults with advanced stages of HIV-infection. We explored T-cell subsets as predictors of mortality and found that they predict death and displace total CD4^+^ count in multivariate models. The nutritional intervention we trialled did not affect these subsets, so we have not yet found a tool for manipulating T-cell subsets or ameliorating the impact of undernutrition on mortality during ART initiation [17]. In our analysis, a high number of naïve CD4^+^ T-cells and a high number of senescent CD8^+^ T-cells at ART initiation were associated with mortality, whilst a high number of proliferating CD4^+^ or CD8^+^ T-cells were associated with survival.

We found a consistent effect of proliferating CD4^+^ and CD8^+^ T-cells on mortality, such that higher levels of proliferation were associated with protection against death. Other studies have shown that CD4^+^ proliferation correlates with viral load; it has been suggested that this is because proliferating (Ki67^+^) CD4^+^ T-cells can efficiently support virus replication [19,20] and lead to more rapid progression to AIDS [21]. In our cohort of malnourished adults, however, the effect was the opposite. Importantly, our cohort was moderately or severely malnourished, and this may be a critical point of difference. We speculate that T-cell proliferative ability reflects a greater ability to respond to co-infections, which are the most frequent cause of mortality after ART initiation [22]. By contrast, a low proportion of proliferating cells in those who subsequently died may signify immune exhaustion, which is known to affect proliferative capacity and may be a pre-terminal event in a malnourished individual. Although we did not evaluate cellular exhaustion markers, other studies have shown elevated T-cell expression of PD-1 [23], CTLA-4 [24] and TIM-3 [25] in advanced HIV disease, which correlate inversely with cellular proliferative capacity.

We also found that higher numbers of senescent CD8^+^ T-cells predict mortality. During HIV infection persistent T-cell activation drives proliferation that results in the accumulation of senescent, antigen-experienced memory T-cells with reduced expression of CD28 and increased expression of CD57 [26]. Expression of CD57 has been linked to greater resistance to apoptosis in CD8^+^ T lymphocytes during HIV infection, facilitating accumulation [27]. Since CD8^+^ T-cells are crucial to the recognition and clearance of virus-infected cells [28,29], a high number of senescent CD8^+^ T-cells may underlie the inability of T-cell immunity to suppress virus adequately.

It has been reported that naïve CD4^+^ T-cells are progressively depleted during HIV infection because of their frequent activation and differentiation into memory cells [30]. A study by Schacker and colleagues found that patients with relatively high numbers of naive CD4^+^ T-cells were less likely to have failure of immune reconstitution with ART and had lower risk of opportunistic infections and mortality [9]. By contrast, low pre-ART naïve CD4^+^ T-cell frequencies predicted poor ART-mediated CD4^+^ T-cell recovery in other studies [31,32]. Additionally, Zhang and colleagues recently showed that increases in naïve CD4^+^ T-cells following ART initiation independently predicted subsequent CD4^+^ T-cell increases [33]. However, our data show that having more naïve cells at baseline increased the likelihood of death during ART in this Zambian cohort with advanced disease. We speculate that predominance of naïve cells at baseline may reflect a permanent loss of important memory cell clones, which cannot be reconstituted despite effective ART. Again, this might be critically dependent on nutritional status (if severe malnutrition imposes a replication constraint on proliferating cell lineages) and/or in a setting where opportunistic infections are highly prevalent. Further studies in different patient groups may clarify these differences.

Although there was a small but significantly greater rise in total CD4^+^ count in the LNS-VM vs LNS group in the main NUSTART trial (difference +25 cells/μL; *p* = 0.02) [17], we found no major differences among T-cell subsets in this sub-study. The only exception was the population of recent thymic emigrants, which increased more in the LNS group than in the LNS-VM group. We speculate that there may be a constraint on proliferation imposed by severe malnutrition and the LNS alone might not be sufficient to permit restoration of thymic output.

This study had several strengths and some limitations. The major strength of this study is the detailed immunophenotyping that was conducted in malnourished HIV-infected adults with advanced disease, which allowed us to address questions related to early mortality, together with the randomized trial design, which allowed us to evaluate the impact of a nutritional intervention on lymphocyte subsets. Study limitations include lack of viral loads, which are not generally available in sub-Saharan Africa for monitoring ART response, and the small sample size at 12 weeks, because of loss to follow-up. Because the participants in this study had advanced disease (median CD4 at ART initiation 98 cells/μL) and malnutrition, our findings may not be generalizable to the larger population of HIV-infected adults initiating ART in sub-Saharan Africa, although it is worth noting that the mean CD4 count at ART initiation in sub-Saharan African programs in 2002 was 152 cells/μL and has not increased significantly since then, meaning that many patients still start ART in the setting of advanced immunodeficiency [34].

In this report of a sub-study of a large clinical trial in adults initiating ART, we found no substantial differences in the effects of a nutritional intervention on specific T-cell subsets, but found that baseline numbers of proliferating CD4^+^ and CD8^+^ cells, naïve CD4^+^ cells, and senescent CD8^+^ cells at ART initiation impacted early mortality on ART. These findings provide insights into the immune dysfunction of AIDS in the context of malnutrition and suggest that baseline immune abnormalities in this population render individuals susceptible to co-infections, which are associated with very high mortality despite ART initiation. Whether patients would benefit from adjunctive infection control interventions at initiation of anti-retroviral therapy (such as additional antiretrovirals and a package of anti-microbial drugs) in addition to a nutritional intervention, is currently being explored in sub-Saharan Africa (REALITY trial; ISRCTN43622374). Our findings also argue strongly for earlier initiation of antiretroviral therapy in sub-Saharan Africa [34], because the immune perturbations that characterize advanced immunodeficiency are difficult to reverse.

## Supporting Information

S1 Table Table for Micronutrient doses in trial supplements, with reference to Recommended Nutrient Intakes for UK women. (PDF)
