# Supplementary material for: T-Cell Subsets Predict Mortality in Malnourished Zambian Adults Initiating Antiretroviral Therapy
Source: PLoS One. 2015 Jun 17;10(6):e0129928. doi: 10.1371/journal.pone.0129928 (PMC4470912; doi:10.1371/journal.pone.0129928)
Supplement: S1 Table — (PDF) [file pone.0129928.s001.pdf]

Supplementary table 1. Nutritional composition of trial supplements – amounts per day[1,2]

Nutrient	First phase supplement (from recruitment to 2 weeks of ART)		Second phase supplement (from 2 to 6 weeks of ART)	
	LNS-VM (30g)	LNS (30g)	LNS-VM (250g)	LNS (250g)
Calories (kcal)	139	168	1397	1416
Protein (g)	2.4	2.3	55	55
Fat (g)	11.0	10.9	97.5	97.5
Potassium (mmol)	30	0.9	32	15.8
Phosphorus (mmol)	47	0.4	38	9.3
Magnesium (mmol)	16	0.3	17	5.7
Calcium (mg)	29.8	5.0	140	115
Iron (mg)	0.4	0.4	14.7	8.4
Zinc (mg)	21	0.2	21	3.8
Copper (mg)	3.6	0.06	3.6	1.2
Manganese (mg)	4.2	-	4.2	-
Iodine (μg)	420	-	420	-
Selenium (μg)	180	-	180	-
Chromium (μg)	75	-	75	-
Retinol (as palmitate) (μg)	1800	-	1800	-
Vitamin D (μg)	10	-	10	-
Vitamin E (mg)	45	-	45	-
Vitamin K (μg)	95	-	95	-
Vitamin C (mg)	120	-	120	-
Thiamin (mg)	2.4	-	2.4	-
Riboflavin (mg)	3.3	-	3.3	-
Niacin (mg)	39	-	39	-

Pyridoxine (mg)	3.6	-	3.6	-
Folate (μg)	600	-	600	-
Vitamin B12 (μg)	4.5	-	4.5	-
Pantothenic acid (mg)	9	-	9	-

[1]Where nutrient contents are provided for both LNS and LNS-VM, these are values from analysis by the manufacturer, accounting for inter-batch variability; where values for only LNS-VM are given, these where not assessed in the prepared foods but refer to amounts added, that is, they do not include those innate to the LNS.

[2]ART=antiretroviral therapy; LNS=lipid-based nutritional supplement, LNS-VM=LNS with added vitamins and minerals
